# Observations and Reflections on the Employment of Strychnine in the Treatment of Paralysis

**Published:** 1833-08

**Authors:** E. Geddings

**Affiliations:** Lecturer on Anatomy and Surgery, Charleston, S. C.


THE LONDON

### Medical and Physical Journal.

414, VOL. LXX.] AUGUST 1833. [86, *New Series.*

"For many fortunate discoveries in medicine, and for the detection of numerous errors, the world is indebted to the rapid circulation of Monthly Journals; and there never existed any work to which the Faculty in Europe and America, were under deeper obligations than to the 'Medical and Physical Journal of London,' now forming a long but an invaluable series."—RUSH.

### ORIGINAL PAPERS.

### STRYCHNINE IN PARALYSIS.

*Observations and Reflections on the Employment of Strychnine in the Treatment of Paralysis.* By E. GEDDINGS, M.D., Lecturer on Anatomy and Surgery, Charleston, S. C. [*American Journal of Med. Sciences.*]

THE materia medica is subject to incessant revolution. Daily experience unfolds to us some new therapeutical resource; and, while hundreds of new remedies, which have been issued into notice by the most extravagant encomiums, are perpetually passing into a speedy and well-merited oblivion, bountiful Providence reveals to us many invaluable properties in others, which we have been accustomed to overlook, or consider as possessing but feeble claims upon our attention. But a few years ago, and the principles upon which the efficacy of Peruvian bark and opium depends were unknown, and those articles could not be divested of the disagreeable concomitants which necessarily attended their administration. But what are the lights of modern chemistry not capable of accomplishing? Guided by its influence, what an inestimable blessing has the ingenuity of Pelletier, Caventou, Sertuerner, &c. conferred upon mankind, by their discovery of the active principles of these articles! a discovery scarcely less important than that of the circulation of the blood. It is moreover but a short time since nux vomica was suffered to moulder upon our shelves; but this neglected substance was destined to furnish, to the researches of Fouquier results capable of leading to the consummation of the most important purposes. This distinguished physician has ascertained that in nux vomica we possess a most efficient means of treating paralysis, a disease which, under all circumstances, is exceedingly difficult to manage, and which, under our former resources, too often baffled our best directed efforts. Chemistry has moreover taught us that these important properties reside, in a concentrated form, in a peculiar substance which has been designated strychnine. It is with a view of adding our feeble weight of testimony to that which has been already furnished in favour of the remedy in the treatment of paralysis, that the following cases have been drawn up. The results which they have furnished have tended much to heighten our faith in the efficacy of the article, and we shall feel pleased if the communication of our own experience should incite others to make trial of it under similar circumstances.

The first case we shall detail is one of hemiplegia of the whole of the left side of the body. The individual was a male, aged about fifty, who was employed as a boat hand. He was placed under our care about the 20th of July, 1829; and, from all the particulars of his case which we were able to collect, it appeared that he had been very suddenly attacked with the disease, about a month previously, whilst engaged in cutting wood. He was seized with violent pain of the head, left arm and leg, fell down in a state of insensibility, to which succeeded in a short time a total loss of sensation and motion of the leg and arm, and double vision. He was still in this condition when we first saw him, with the exception that the double vision had disappeared, and he was so far sensible, as to reply rationally when addressed in a loud voice. There was no febrile disturbance, no impediment in the powers of speech, and the principal difficulty consisted in a loss of sensation and motion on one side. The leg and arm could be violently pinched or pricked without the patient evincing the slightest feeling, and the only pain he complained of was in the head and back of the neck.

He was ordered an active cathartic of calomel combined with jalap, principally with a view of creating a revulsive impression from the head to the lower intestines. Directions were also given to have his head shaved and blistered, and to have sinapisms applied to the legs.

21st. The medicine has operated, and the blister has drawn well, but without producing any improvement in the condition of the patient, except a slight amelioration of the pain of the head and neck. He is restless, and his mind somewhat wandering.

22d. The same condition. Cathartic directed to be repeated, and cups to be applied to the nape of the neck, the part afterwards to be covered by a blister.

23d. No improvement. Ordered sinapisms to the legs, and stimulating frictions to the paralytic members.

24th. The same condition continuing without any amelioration, we resolved to put him upon the use of the strychnine. The following was therefore directed: ℞. Strychnine, gr. vj.; alcohol, ℥i.; of which six drops were administered morning and evening.

25th. The dose of the medicine was increased to ten drops twice a day.

26th. Experienced this morning considerable spasmodic action of the muscles of the paralytic members. Mentioned the circumstance without being questioned, and compares the sensation to that which might be occasioned by living animals leaping in the flesh. No affection of any but the paralyzed muscles. Complains for the first time of pain when pricked with a pin. Medicine to be continued.

27th. Slight improvement. Complains of pain in the back of the neck. Ordered a blister to be applied to that part; tincture of strychnine to be increased to fifteen drops.

28th. Pain relieved. Can flex the leg slightly without assistance. The sensibility is also improved. Has had twitchings of the muscles of the arm. The paralytic leg and arm have become œdematous. Medicine to be continued.

29th. The improvement is progressive. The leg when pricked suddenly was flexed with great force. Arm, however, in nearly the same state.

30th. Can flex the leg to a right angle with the thigh, and again extend it. Has had twitchings of the arm and leg. The same prescription continued.

31st. Further improvement. Can flex and extend the leg with great freedom. No perceptible improvement of the arm.

August 1st. The improvement is progressive. Medicine to be increased to eighteen drops three times a day.

2d and 3d. Improvement continues. The muscles of the arm affected still more with spasm. Medicine increased to twenty-four drops.

4th. The condition of the leg continues to improve. The arm, however, remains in nearly the same state. From this time the amelioration was uninterrupted; and at the expiration of about a week he began to walk with the assistance of a crutch, and in a short time the power of motion in the leg so far improved as to enable him to walk to any part of the city. He finally recovered sufficiently to be sent into the country. At the time of his departure, however, the arm had not regained its powers, and had undergone but a very slight amelioration. Since then we have not been able to learn any thing of his situation.

This case is calculated to furnish some very important deductions relative to the administration of strychnine in cases of paralysis. From the general phenomena exhibited during its progress, as well as the nature of the attack at first; the complete loss of sensation, succeeded almost immediately by a total loss of motion, on one entire side of the body; the mental imbecility and wandering, which continued even throughout the whole course of the disease, all conspire to render it almost certain that the hemiplegia originated from an extravasation of blood in, or upon, some part of the brain.

This was indeed our opinion of the case from the period it first came under our attention. Under these impressions, the indications seemed to be to subdue local irritation by cups and blisters, and to create a revulsive impression from the brain to the bowels and extremities, by means of drastic cathartics, and sinapisms applied to the legs. We had always thought, with Andral and others, that nux vomica, strychnine, or brucine, could not be used with any benefit in cases of paralysis arising from a recent attack of apoplexy. We had even publicly deprecated the practice, in our lectures, as fraught with danger. It was therefore only after we had used the other means, without any apparent benefit, and had, from the general aspect of the case, considered it one of a desperate character, that we resolved to give the strychnine a trial, not with any hope of advantage, but principally with a view of testing its effects upon the constitution, under similar circumstances. The results disappointed our expectations, and have tended somewhat to modify the opinion we had advanced. We are however far, even now, from considering the remedy appropriate to the generality of cases of paralysis proceeding from recent attacks of apoplexy. On the contrary, we still think that, in a majority of them, it would not only fail of success, but would prove prejudicial, if used before the proper preparation had been made by depletion. What, in effect, is the condition of the brain under such circumstances? It is at first compressed by the extravasated blood, and this compression is of itself sufficient to give rise to paralysis. But, in addition to this, the extravasated blood, acting upon the principle of a foreign body, produces a constant source of irritation to the adjoining portion of the brain; an increased determination of blood takes place to the point; its nutrition becomes modified, its texture softened, and its cohesive power broken up. This condition cannot therefore fail to augment the paralysis; and, while it continues, we can hope nothing from the stimulating influence of strychnine. Direct depletion and revulsion are the grand hinges upon which the whole of our treatment must turn, and by no other procedure can we hope to relieve our patient. But, in process of time, the condition of the organ becomes, by a series of changes, very much altered. We know, from attending to the pathological condition of the tissues and organs, that a speedy effect of irritation is to give rise to a ramollissement, or softening, more or less extensive. We also know that, after a time, under certain modifications of the same process, new molecules are elaborated, coagulable lymph is deposited in the midst of the pre-existing molecules, the rudiments of vessels permeate this new product, it becomes organized, grows gradually more and more compact; and, in cases of apoplexy, finally forms a complete cyst, or membrane, surrounding the extravasated blood; thus completely isolating it from the brain, and cutting off or counteracting its injurious influence upon that organ: until this is effected, a link in the chain of influence between the brain and a portion of the nerves is broken; and, as the latter derive their controlling power from the former, it necessarily follows that, while this severed link remains unrepaired, a paralysis more or less extensive must exist. Strychnine, under such circumstances, cannot repair the breach, and therefore has no power to remove the influence which it exercises upon the muscles. But so soon as the resources of the living organization have, by setting up the changes which have been pointed out, restored to a certain extent the injury which has been sustained by the brain, the parts have acquired a condition which warrants us in hoping something from the stimulating influence of that article. But it may be asked, why is there any necessity for resorting to such adventitious aid after the lesion of the organ has been repaired? We would reply, for two reasons: 1st. That the integrity of the organization is not completely restored; consequently, we cannot expect that the integrity of its operations will be renewed without assistance. 2d. That, from the long quietude, or repose, to which the paralytic muscles, or rather nerves, have been submitted, the brain does not, when restored, regain immediately its controlling influence over them, which it only retains by degrees, and by the aid of some agent which possesses the power of exciting in a special manner the nerves themselves, so as to exalt their vital properties to that degree which is essential to enable them to feel and act under the influence of the brain. Do we not, in effect, sometimes observe the muscles, even where the brain possesses its perfect integrity, lose their power of acting, merely from long-continued repose? Do we not find them becoming relaxed, wasted, and enfeebled, in the inactive and sedentary, merely from the want of employment? This can only be ascribed to the controlling agency of the brain becoming weakened, because it is not exercised, or to the susceptibility of the nerves to the empire of that organ being diminished, from their being suffered to remain for a length of time in a quiescent or inactive state. The parallel, we think, holds good with paralysis; and, under the circumstances pointed out, the efficacy of strychnine, in restoring the powers of sensation and motion, depends entirely on its peculiar property of exciting the nerves, and thus exalting their vital powers to that degree which is requisite to enable them to feel and react under the empire of the great nervous centre. If these views be correct, they fully explain to us the futility, or even danger, of administering the article in question in that form of paralysis which succeeds apoplexy, before sufficient time has elapsed for the changes which have been pointed out to effect the restoration of the part, and the good effects which we sometimes have reason to hope from it, after they have produced their full effect.

Another phenomenon was observed in this case, as it has been in many others,—the limitation of the tetanic contractions of the muscles to the paralytic members. At no period of the disease was any thing of the kind observed on the opposite side. The same was true of an œdematous affection which took place in the paralysed leg and arm, shortly after we began the administration of the strychnine. It would be difficult to offer a satisfactory explanation of either of these phenomena. We cannot suppose that the medicine could exercise an exclusive influence upon the paralysed nerves, without affecting those which are in a healthy state. It must operate upon both in an equal degree; and we can afford no more satisfactory explanation of the spasmodic action in question, than upon the supposition that the nerves become unusually excited, while the actions which they communicate to the muscles are deprived of the regulating influence of the brain, which we have seen is cut off in the manner above pointed out: hence they are thrown into irregular, convulsive motions, while the healthy muscles act regularly. But it may be urged against this hypothesis, that the healthy, as well as the diseased muscles, sometimes experience the same irregular actions. In reply to this objection, we would state that, under such circumstances, something has transpired to break up or derange the natural harmony which directs or regulates the controlling influence of the brain over the nerves, so that the natural susceptibility of the one or the other is modified, or that some irregularity takes place in the transmission of the influence of the one through the other; and, consequently, irregular discordant contractions are the necessary result. How often indeed do we find the muscles, even in the healthy state, becoming afflicted with violent cramps or uncontrollable tremors, from over-exertion or fatigue? and is it not manifest that, under such circumstances, some derangement of the kind to which we have adverted takes place? We are free to confess that we can offer no other solution of the question which is at all satisfactory to ourselves.

As to the œdema, it is a question whether it should be considered as a consequence of the paralysis, or of the operation of the strychnine. We can readily conceive how a paralysis of the muscles may, by depriving the veins of that presence and support which is partly instrumental in propelling the blood through them; and how the venous trunks, by becoming thus distended, may, by being unable to receive and circulate their usual quantum of fluid, occasion the œdema of the members. But in the case before us, as well as in one or two others which we have witnessed, although the paralysis had existed for a length of time, (in one more than a year,) no œdema made its appearance until after the strychnine had been administered. Supposing then the medicine to have been instrumental in producing it, what rationale can we offer of the fact? There are some other articles of the materia medica (for instance, arsenic) which possess the power of producing similar serous effusions. It is difficult, however, to conceive how any of these articles can produce such effects. Can it be by enfeebling the action of the heart and arteries? We think not; for in many cases a febrile condition exists, with frequent, hard, and strong pulse. Is it by altering the condition of the capillary vessels, and modifying their operation, so as to destroy the harmonizing relation which exists between the arterial capillaries, by which the fluids in question are exhaled, and the venous and lymphatic radicles by which they are absorbed? This we consider a more rational solution of the question, inasmuch as we have generally observed that the administration of the medicine is productive of great pain and soreness of the common integuments, which frequently continues long after it has been left off. Irritation of the capillaries, produced by the influence of the strychnine, is therefore the only rational explanation we can offer of the phenomena in question. But this does not by any means explain why it is that the œdema is confined to the paralytic members. We can only offer a conjecture upon this point,—that the capillaries being irritated, and, with the veins, deprived of the support afforded by the surrounding muscles, œdema will take place more readily than where the muscles act, and, by the compression they exercise upon these vessels, propel the fluids through them.

The next case we shall detail is one of a very different character.

Thomas Wood, aged twenty-eight years, was admitted into the Charleston Alms-house Infirmary, on the 18th of October, 1829, labouring under general paralysis. Of his precise condition at the period of his admission we are not acquainted, as we were not at that time attached to the institution. One thing however is certain, that he was completely helpless, and we believe his general health was very much impaired, from the intemperate use of ardent spirits, and the exposures and privations incident to his habits. About the middle of January, 1830, when he first came under our observation, his general health had improved considerably under the treatment to which he had been submitted, but the paralysis had undergone but little or no amelioration. His legs and arms were so completely powerless as to render him incapable of helping himself, and whatever he took by way of food or medicine had to be put into his mouth by another person. His legs were drawn up to a right angle with the thighs, and could not be straightened. His bowels had moreover so far lost all power as to be incapable of expelling their contents, except when inordinately excited by stimulating enemata. The state of the patient could not be attributed to any thing like extravasation within the cranium, as he never had suffered any thing which could lead to a suspicion of such an occurrence. His previous habits, as well as the general aspect of the case, furnished a strong presumption that it originated from the long-continued and intemperate use of bitter beer and ardent spirits, especially gin, which had probably been adulterated with acetate of lead. That this was the case we are disposed to infer, independently of other considerations, from the fact, that we have met, in hospital practice, and amongst the poor, with a number of cases of paralysis, and have generally found that the subjects of these attacks have been persons addicted to the immoderate use of inferior gin and beer. These cases have, moreove, all presented the usual manifestations of paralysis from lead.

Taking this view of the case, we resolved to try the effects of strychnine, administered in liberal doses. He was accordingly directed to have daily a strong aloetic injection for the purpose of unloading the bowels, to be followed, after the operation was over, by half a grain of strychnine, suspended in a small quantity of mucilage. He was also directed to take the twelfth of a grain of the same medicine night and morning. This treatment was instituted on the 15th of January, and was continued until the 24th. On the 16th a splint similar to Pemberton's was applied to the left arm by way of experiment, but, after being worn for some time, it appeared to be rather prejudicial than advantageous, and was consequently omitted.

24th. The strychnine, in the doses above mentioned, was directed to be given thrice a day. The aloetic injection and the half-grain of strychnine to be continued as before. On the 26th the medicine was increased to the sixth of a grain night and morning. The compound aloetic pills were also directed every night, in sufficient doses to excite the lower bowels. Strychnine injection still continued. No further alteration was made in the treatment until the 8th of February, when the medicine was increased to three pills, of the twelfth of a grain each, night and morning. The aloetic pills and strychnine injection to be continued as above. A carved splint, long enough to extend from the nates to the heel, and also bound upon the posterior part of the thigh and leg, on one side, with a view of overcoming the flexed position of the member. After using it, however, for several days, without any benefit, it was abandoned. On the 16th, in addition to the other remedies, we directed the muriated tincture of iron with a view to its tonic effect, and it, with the other remedies, was continued until about the middle of March.

For several days after the strychnine was first administered, very little change was observed in the condition of the patient, and the usual tetanic contractions were scarcely observable; nor were they considerable at any period during the treatment. In the course of a fortnight a marked improvement was observable. The patient began to flex and extend his arms with facility, and could grasp any thing in his hands with considerable firmness. From this time, his improvement was progressive, but slow. His bowels soon began to act with more freedom; his ability to move his limbs increased, and in a short time he so far recovered the use of his arms as to turn and move himself in bed, and take his food and drink without assistance. By the 1st of March the mobility of the members had become so far restored, that nothing seemed to prevent him from walking but the flexed position of the legs and a want of confidence in his own powers. These difficulties were gradually surmounted by means of frictions and exercise; and at the present time, though he still experiences a little stiffness of the members, he can walk without assistance, and descends and ascends almost daily a long flight of stairs. The bowels have been for some time past in possession of their natural tone, and evacuate their contents without the aid of medicine. The general health of the patient is entirely restored.

This case exhibits to us many features of an important character: the cause by which it was produced; the great extent of the paralysis, rendering the patient completely helpless, and especially the implication of the muscles of organic life, as exemplified in the paralysis of the rectum, are considerations which deserve comment.

We have already stated our reasons for supposing the disease to have been produced by lead, employed in the adulteration of the liquor which the individual had been in the habit of using immoderately. The operation of this cause was probably assisted by the exposure, the irregular diet, and other accidental circumstances to which his intemperate habits exposed him. But we are here met by a question of considerable interest: how do the salts of lead operate in producing this paralysed condition of the muscles? To this it is not an easy matter to reply satisfactorily, in the present state of our knowledge. One thing however is certain, that these preparations exercise an influence directly debilitating upon the animal organization; or, in other words, that they diminish the nervous susceptibility, and thereby diminish or modify irritation in the nerves themselves, as well as in all the tissues to which they are distributed. If, with the Italian school, we divide all agents into stimulants and counter-stimulants, lead would fall under the latter class; a situation to which its effects on the constitution clearly entitles it. If we are asked for proofs of this, we will only reply, that they are furnished in great abundance in the daily employment of the article in the treatment of diseases, in its soothing influence upon inflamed surfaces, in its power of controlling the exalted irritation of the mucous membrane in fever, in moderating bronchial irritation in chronic catarrh and phthisis pulmonalis, in arresting hemorrhage, &c. It is therefore in virtue of a similar operation upon the nervous and vascular systems, long continued, and carried to an inordinate extent, that the salts of lead occasion paralysis. This it may effect by being taken into the stomach, inhaled by the lungs, or applied to the skin. In either case its action seems to be exercised directly upon the nervous filaments which preside over the functions of the several tissues to which it is first applied. We shall endeavour to illustrate our proposition by considering those cases in which the lead has been taken into the stomach.

The stomach and intestines derive their nerves from two sources or systems; the one ganglionic, coming from the great sympathetic, and subservient to circulation, secretion, &c.; the other from the pneumogastric, destined more especially to preside over the contractions of the muscular coat of the tube. These form a free and intimate connexion with the spinal and other nerves, by means of numerous anastomosing branches; so that whatever affects the one must have its influence not only extended to the others, but even to the spinal marrow, and the parts within the cranium. Lead therefore applied to the mucous surface of the alimentary canal, and exercising upon it a directly counter-stimulant impression, two important consequences are immediately developed: 1. The nervous filaments which regulate contractility, circulation, secretion, &c. are debilitated; the capillaries, deprived of their nervous influence, are unable to propel their contents forwards; a congestion consequently takes place in these vessels, the harmony of the circulation is thus deranged, and the secretions diminished, suspended, or perverted. 2. The filaments which control the muscular coat being submitted to a similar impression, that tunic becomes enfeebled or paralysed, and, when these conditions are developed in a considerable degree, all the phenomena of colica pictonum are manifested. By an extension of this influence still further, consequences of a still more formidable character are produced. Where the impression made by the counter-stimulant influence of the lead is intense, or when it has been maintained for a length of time, it is finally communicated to the spinal marrow, which thus having its powers enfeebled or paralysed, the parts which it, as a great nervous centre, controls and regulates, must suffer in a proportionate degree. This we consider as the train of sequences which attends the formation of paralysis from lead; an opinion which seems to be justified as well by the phenomena of the disease, as by the state of the intestinal tube in those who have died affected with that malady. What, in effect, are the first symptoms which arise from the employment of the article in question? The secretions are suspended, and the muscular coat of the intestine is paralysed; hence an obstinate constipation of the bowels ensues. To this succeeds pain, because the congestion of the capillaries, occasioned by the diminution of their contractile or propulsive energies, together with the retention of the indurated fæces, give rise to irritation, which, in many cases of colica pictonum, is strongly marked. Finally, the nervous centre becomes affected, and from it the influence is extended to the muscles of animal life, which become enfeebled or paralysed. This view of the pathology of the disease not only comports best with its phenomena, but also with the results obtained in its management. It has been most satisfactorily ascertained that those remedies are most successful in the treatment of paralysis from lead which exercise a powerful, stimulating impression upon the nervous system, as electricity, galvanism, frictions, nux vomica and its preparations, brucine, rhus toxicodendron, arnica, &c. This was strongly exemplified in the second case, already detailed. We have seen that the paralysis not only implicated the muscles of animal life, but that the lower bowels were so much affected as to render them incapable of performing their office, except when excited by powerful stimuli. It was under these circumstances that the half-grain of strychnine was administered by injection, with a view of acting directly upon the affected part. Under its employment the contractility and secretions of the tube were gradually restored; and, in proportion as this restoration took place, the intestine regained its wonted power. The influence of the medicine, moreover, which was administered by this process and by the mouth, was next communicated to the spinal marrow, or nervous centre, and from thence to the nerves which supplied the paralytic muscles, which, being invigorated by this new impression, were restored to their former power. This is the exposition of the phenomena of the cure and its treatment, which, after watching its progress, and reflecting upon the circumstances attending it, we feel ourselves warranted in making. Whether our views be correct or not, the different articles which have been mentioned are certainly the most efficacious that can be administered in this form of paralysis; and, under proper management, we believe that, in such cases, it will seldom disappoint our expectations. In proof of its efficacy we might adduce several other cases, less formidable in their nature, but originating in similar habits, and under similar circumstances.

John Hood, aged thirty years, was admitted into the Almshouse Hospital, on the 8th of June, 1830, affected with paralysis of the forearm and hand. The powers of motion were almost entirely destroyed, and sensation was so far extinct that the part could be severely pinched without occasioning pain. He took the strychnine in the doses employed in the preceding cases, and, by persisting in its use for about a fortnight, using at the same time frictions and pumping, he was relieved. The habits of this individual were intemperate; and it is highly probable that the affection of the arm proceeded from the influence of lead employed in adulterating the liquors which he drank.

Daniel Reardon, aged fifty-two years, came into the hospital on the 20th of July, 1830, with the forearm and hand paralysed, and was put upon the same course of treatment, which afforded speedy relief. At present he moves his arm and hand with great freedom, and only complains of a slight degree of weakness in the part. His habits are intemperate, and he states that he has been accustomed to drink very freely of common northern gin. We can assign no other cause for his disease than that which has been offered in the above cases.

In conclusion, we think it is undeniable that, in the above cases, the good effects of the strychnine were strikingly manifested, and that, under similar circumstances, much may be expected from the judicious employment of that article. We are at present using it in some other cases, but are unable as yet to determine what will be the result. We will take this opportunity of observing, that we have employed strychnine with much apparent benefit in some forms of chronic irritation of the alimentary canal, especially in persons who were affected with habitual constipation of the bowels. It should, however, always be borne in mind that its natural tendency is to irritate the mucous membrane.

---

